# Functional connectivity-hemodynamic (un)coupling changes in chronic mild brain injury are associated with mental health and neurocognitive indices: a resting state fMRI study

**DOI:** 10.1007/s00234-024-03352-9

**Published:** 2024-04-12

**Authors:** Antonios Kagialis, Nicholas Simos, Katina Manolitsi, Antonios Vakis, Panagiotis Simos, Efrosini Papadaki

**Affiliations:** 1 Department of Psychiatry, School of Medicine, University of Crete, University Hospital of Heraklion, Crete, Greece; 2 Department of Radiology, School of Medicine, University of Crete, University Hospital of Heraklion, 71003 Crete, Greece; 3 Institute of Computer Science, Foundation for Research and Technology – Hellas, Heraklion, Crete, Greece; 4 Department of Neurosurgery, School of Medicine, University of Crete, University Hospital of Heraklion, Crete, Greece

**Keywords:** mTBI, Rs-fMRI, ICC, TSA, Neurovascular (un)coupling

## Abstract

**Purpose:**

To examine hemodynamic and functional connectivity alterations and their association with neurocognitive and mental health indices in patients with chronic mild traumatic brain injury (mTBI).

**Methods:**

Resting-state functional MRI (rs-fMRI) and neuropsychological assessment of 37 patients with chronic mTBI were performed. Intrinsic connectivity contrast (ICC) and time-shift analysis (TSA) of the rs-fMRI data allowed the assessment of regional hemodynamic and functional connectivity disturbances and their coupling (or uncoupling). Thirty-nine healthy age- and gender-matched participants were also examined.

**Results:**

Patients with chronic mTBI displayed hypoconnectivity in bilateral hippocampi and parahippocampal gyri and increased connectivity in parietal areas (right angular gyrus and left superior parietal lobule (SPL)). Slower perfusion (hemodynamic lag) in the left anterior hippocampus was associated with higher self-reported symptoms of depression (*r* =  − 0.53, *p* = .0006) and anxiety (*r* =  − 0.484, *p* = .002), while faster perfusion (hemodynamic lead) in the left SPL was associated with lower semantic fluency (*r* =  − 0.474, *p* = .002). Finally, functional coupling (high connectivity and hemodynamic lead) in the right anterior cingulate cortex (ACC)) was associated with lower performance on attention and visuomotor coordination (*r* =  − 0.50, *p* = .001), while dysfunctional coupling (low connectivity and hemodynamic lag) in the left ventral posterior cingulate cortex (PCC) and right SPL was associated with lower scores on immediate passage memory (*r* =  − 0.52, *p* = .001; *r* =  − 0.53, *p* = .0006, respectively). Uncoupling in the right extrastriate visual cortex and posterior middle temporal gyrus was negatively associated with cognitive flexibility (*r* =  − 0.50, *p* = .001).

**Conclusion:**

Hemodynamic and functional connectivity differences, indicating neurovascular (un)coupling, may be linked to mental health and neurocognitive indices in patients with chronic mTBI.

**Supplementary Information:**

The online version contains supplementary material available at 10.1007/s00234-024-03352-9.

## Introduction

Traumatic brain injury (TBI) represents a major health and socioeconomic problem worldwide [1]. The vast majority of TBI cases are classified as mild (mTBI), with a Glasgow Coma Scale (GCS) of 13 or higher [2]. It is estimated that 15–30% of patients with mTBI present with persistent symptoms long after initial trauma [3], such as cognitive impairment [4] and emotional difficulties, including depression [5] and anxiety [6]. Although CT is the imaging modality of choice in acute TBI and should be used in certain cases of mTBI, it is often normal in these patients and holds a poor prognostic value [7]. Conventional MRI reveals more subtle injury compared to CT and improves short-term outcome prediction [8], but it is also normal in many mTBI cases because it fails to reveal microstructural, hemodynamic, and functional alterations. On the contrary, advanced neuroimaging techniques (i.e., perfusion imaging and functional MRI) provide quantitative estimation of vascular and functional abnormalities and could provide a better understanding of mTBI pathophysiology and more accurate prediction of long-term outcomes in these patients [9].

Hemodynamic abnormalities in several brain regions of patients with chronic mTBI have been described by using perfusion MRI techniques (i.e., Dynamic Susceptibility Contrast MRI (DSC-MRI) and Arterial Spin Labeling (ASL)). These regions include bilateral frontotemporal lobes [10], superior temporal cortex [11], left dorsal anterior cingulate cortex [12, 13], and the cuneus, middle temporal gyrus, and cerebellum [13]. We have recently reported significant hypoperfusion in dorsolateral prefrontal areas, putamen, and hippocampus bilaterally [14] in patients with chronic mTBI. In that study, depressive symptomatology was significantly associated with lower perfusion in the left anterior cingulate gyrus, while severity of anxiety symptoms was associated with lower perfusion in the hippocampus.

Resting-state fMRI (rs-fMRI) studies in patients with mTBI have shown functional connectivity (FC) alterations between and within intrinsic brain networks, including the default mode network (DMN) and fronto-parietal, motor, dorsal attention, and visual networks [15–21]. Reduced FC within the DMN has been reported in patients with acute/subacute mTBI, linked to neurocognitive dysfunction and severity of posttraumatic symptoms [16, 18–25]. Furthermore, altered connectivity between the DMN and other networks has been shown in chronic mTBI [15, 26], while abnormal connectivity of the DMN with task-positive and salience networks was linked to poorer memory performance [27]. In a recent study of our group, machine learning, and graph theory were used to combine static and dynamic FC data of patients with chronic mTBI. Hypoconnectivity was displayed in the temporal poles, which correlated positively with semantic and phonemic verbal fluency, while hypoconnectivity in the right dorsal posterior cingulate cortex (PCC) correlated positively with depression severity. Conversely, hyperconnectivity was observed in the right precentral and supramarginal gyri, which correlated negatively with semantic verbal fluency, indicating a potentially ineffective compensatory mechanism [28].

A very important sequelae of brain trauma is the injury of the neurovascular unit (NVU), which represents the multicellular structural and functional relationship between the brain and blood vessels and is responsible for the maintenance of the blood–brain barrier (BBB) and cerebral homeostasis, as well as the control of cerebral blood flow (CBF) [29]. Injury of the NVU may occur even after mTBI and lead to ongoing hypoperfusion and neurodegeneration [30]. Neurovascular coupling refers to the mechanism that links transient changes in neural activity to the subsequent change in CBF, and the NVU has a crucial role in this process [31]. Alterations of this “neurovascular coupling” can impair the ability of the brain to provide sufficient blood to active regions, leading to neural dysfunction, and may underlie some of the pathophysiologic mechanisms associated with posttraumatic cognitive and emotional symptoms in TBI [32]. Animal studies indicate that moderate to severe TBI can lead to a reduction in local CBF, resulting in a condition known as neurovascular “uncoupling.” These changes are believed to primarily arise from disruptions in neural regulation and endothelial function within the pial vasculature [33]. Endothelial dysfunction may also underlie impairment in neurovascular coupling after mTBI in humans [34]. Retired boxers can show evidence of cerebral hypoperfusion coupled with neurocognitive dysfunction [35]. However, the link between changes in neurovascular coupling and neuropsychological symptoms in chronic mTBI patients has not been explored.

Rs-fMRI could provide evidence not only about neural activity but also about regional hemodynamic status and neurovascular coupling through time-shift analysis (TSA). TSA is a promising new method that has been used to assess hemodynamics in previous studies [36–46]. According to this method, hemodynamic transfer speed is indexed by the degree of temporal shift of low-frequency BOLD signal fluctuations [45, 46]. A disturbance of local blood flow is reflected in these fluctuations as a localized delay (i.e., hemodynamic lag) or temporal gain (i.e., hemodynamic lead) in relation to the blood flow in major cerebral veins. Substantial shifts in the order of seconds have been shown to provide information about local brain hemodynamics, like established MR perfusion techniques [39, 40, 46].

To date, this technique has been applied not only to neurological conditions characterized by severe perfusion disturbances, such as stroke [36–40], but also in patients with Alzheimer’s dementia or mild cognitive impairment [41], systemic lupus erythematosus [42, 43], clinically isolated syndrome and multiple sclerosis [44], displaying more subtle hemodynamic impairment. By utilizing TSA on rs-fMRI data, it becomes possible to combine hemodynamic and functional connectivity metrics and assess the degree of their coupling (or uncoupling) within specific regions of the brain with the same technique and without the use of contrast agents. Such interdependencies between hemodynamic and functional connectivity changes may reflect pathologic alterations of the NVU and subsequent functional or dysfunctional compensatory processes that develop regionally. Brain damage in mTBI is commonly related to hemodynamic and functional connectivity changes, leading to cognitive and emotional disturbances, even at the chronic stages of the disease. However, studies on alterations in neurovascular coupling and its relationship to cognitive performance and emotional status after mTBI are scarce, and the hemodynamic-connectivity interdependencies in these patients are worth examining.

The aims of the current study are (a) to explore hemodynamic, functional connectivity, and neurovascular coupling/uncoupling alterations in patients with chronic mTBI by using rs-fMRI via TSA and (b) to correlate these changes with the severity of depression and anxiety symptomatology, as well as, with the cognitive function of these patients.

## Materials and methods

### Participants

The present study included 37 patients with chronic mild TBI and 39 healthy controls (HCs). Initially, 46 patients meeting inclusion criteria were identified through the registry of the Neurosurgery Clinic, Heraklion University Hospital, and invited to return for follow-up MRI and neuropsychological assessment. Inclusion criteria were: (a) age at the time of injury, 19–65 years, (b) non-penetrating injury that did not require neurosurgical intervention, (c) mild injury severity as indicated by Glasgow Coma Scale (GCS) score ≥ 13 upon admission [47] and (d) time elapsed since brain injury ≥ 6 months. Exclusion criteria were as follows: (a) history of neurological or psychiatric disease prior to injury, current history of substance abuse, or systematic psychiatric or psychological interventions post-injury or currently receiving psychoactive medications other than anticonvulsants; (b) posttraumatic multifocal or unifocal extensive lesions (i.e., gliotic areas due to contusions > 3 cm or multiple (> 3) chronic hemorrhagic foci resulting from diffuse axonal injuries (DAIs)) at the MRI exam performed at the time of inclusion. Nine patients did not meet the inclusion criteria and were not included in the analyses. None of the patients were involved in litigation concerning their injury or indicated that the results of the study could be used to seek compensation. All HCs underwent a structured interview to record basic demographic information and ensure that they did not meet the exclusion criteria (history of neurological (including TBI) or psychiatric disease, current history of substance abuse, or currently receiving psychoactive medications).

The study was approved by the University Hospital of XXX Ethics Review Board, and details of the procedure were explained to all participants, who provided and signed a written informed consent.

### Neuropsychological assessment

The cognitive and emotional status of all patients with mTBI was assessed on the same day as the MRI session, using a battery of standardized tests available in Greek. Tests covered a wide range of cognitive domains in view of the reported heterogeneity of patient neurocognitive profiles, especially in the chronic phase [48]. The following tests were administered: Memory for Digits Forward and Reverse subtests of the Greek Memory Scale [49] to assess short-term and working verbal memory; The Passage Memory subscale of the Greek Memory Scale and delayed reproduction of the modified Taylor Complex Figure test (TCF) [50] to assess secondary episodic memory. The Trail Making Test (TMT) Parts A and B were used to assess visuomotor coordination speed and mental flexibility [51]. Semantic (SVFT) and phonetic (PhVFT) subtests of the verbal fluency test were employed for the assessment of strategic rule-based access to stored lexical representations [52]. The matrices subtest of the Wechsler Adult Intelligence Scale (WAIS-IV) indicated problem-solving ability [53]. All neuropsychological measures were converted to z scores based on Greek population norms (adjusted for age and education). Furthermore, the Greek adaptations of the Center for Epidemiology Studies Depression Scale (CESD) [54] and Spielberger Trait Anxiety Inventory (STAI-B) [55] were used for the assessment of depression and anxiety symptoms.

### MR imaging

All participants underwent brain MRI scans at the MRI Unit, University Hospital of XXX, using identical scanning parameters. MRI scans were acquired on a clinical, upgraded 1.5 T whole-body superconducting imaging system (Vision/Sonata, Siemens/Erlangen) equipped with high-performance gradients (gradient strength: 40 mT/m, slew rate: 200 mT/m/ms) and a two-element circularly polarized head array coil (minimum voxel dimensions: 70 μm × 70 μm × 300 μm). The main imaging protocol consisted of a 3D T1-w MPRAGE (TR/TE: 1570/1.73 ms, 1 mm/1 NEX/160 axial sections), a T2wTSE (TR/TE: 5000/98 ms, 4 mm axial sections), a Turbo FLAIR (TR/TE/TI: 9000/120/2320 ms, 4 mm axial sections) sequence and a T2*GRE sequence. Axial sections were acquired parallel to the plane, passing through the anterior and posterior commissures (AC–PC line). Structural MR images were interpreted by a senior neuroradiologist, blinded to the clinical and laboratory data, who reported any incidental findings not related to mTBI, such as acute or old infarcts, hemorrhages, and focal brain atrophy, to apply the exclusion criteria listed in Sect. 2.1. Rs-fMRI data were acquired using a T2*-weighted, fat-saturated 2D-FID-EPI sequence with repetition time (TR) 2320 ms, echo time (TE) 50 ms, field of view (FOV) 192 × 192 × 108 (x, y, z). Whole brain 3D images consisted of 36 transverse slices with 3.0-mm slice thickness and no interslice gap. The voxel BOLD time series consisted of 150 dynamic volumes, while the voxel size was 3 × 3 × 3 mm (3 mm isotropic). Initial fMRI data preparation steps are in line with previous work of our team on data from the same MRI system [28, 42–44, 56, 57] (see supplementary material).

### Time-shift analysis

Time-shift analysis maps were calculated as described in several previous studies [36–44] using MATLAB scripts (see also supplementary material). Firstly, a mask of the major venous sinuses was created based on the standard brain. The reference BOLD time series was calculated as the mean of all the voxel time series included in the venous mask. Then, voxel-wise cross-correlations were calculated in reference to this regressor for lags of − 3 TRs to 3 TRs (− 6.96 to 6.96 s). This entails the computation of the lagged versions of each voxel time series (− 3 TR to 3 TR) and of the correlation coefficient of each lagged version of the time series with the reference signal. The lag value corresponding to the highest correlation coefficient was assigned to each voxel as its time shift value. Only cortical voxels included in the 232 regions defined in the Schaefer atlas were considered. The dependent variables used for statistical comparisons and correlations with neuropsychiatric data were the average positive (hemodynamic lag) or negative (hemodynamic lead) delay. Analyses on two complementary indices—percentage of voxels displaying hemodynamic lag (indicated by TSA values > 1 TR of the venous mask average) and percentage of voxels displaying hemodynamic lead (indicated by TSA values < 1 TR)—were also performed to take into account inhomogeneities in voxel distributions within each region.

### Voxel-wise functional connectivity

Voxel-wise global connectivity was assessed through the intrinsic connectivity contrast (ICC), an estimate of the degree of association between the time-series of a given voxel with all other voxels included in the 232 regions of the Schaefer atlas. ICC is based on the graph-theoretical measure of degree (i.e., the number of nodes connected to a given node). A voxel’s ICC value is computed as the mean of that voxel’s time series correlation values with all other voxels’ time series squared. This analysis computes whole brain indices of cortico-cortical functional associations, considering individual variability in connectivity patterns instead of relying on a priori-defined FC networks. Here, the percentage of voxels with ICC values greater than the 75th percentile of all cortical voxels of a given participant and the percentage of voxels with ICC values smaller than the 25th percentile served as dependent variables. The MATLAB code for calculating ICC values using singular value decomposition is freely available online (see supplementary material).

### Voxel-wise TSA-ICC conjunction masks

Four TSA-ICC conjunction voxel masks were calculated by overlaying the following pairs of binary masks for each participant: (i) positive TSA and low ICC (values below the 25th percentile of the distribution of values across all brain regions) indicating FC-hemodynamic coupling in the presence of low FC, (ii) negative TSA and high ICC (values above the 75th percentile) indicating FC-hemodynamic coupling in the presence of high FC, (iii) negative TSA and low ICC indicating FC-hemodynamic uncoupling in the presence of low FC and, ( iv) positive TSA and high ICC indicating FC-hemodynamic uncoupling in the presence of high FC.

It should be noted that all region masks encroach into the white matter directly beneath the cortical mantle. Since all patients presented with no or very mild cerebral atrophy, no normalization to grey matter volume was performed.

### Statistical analysis

The first research aim of the study was addressed via two-sample t-tests on each set of ROI-level indices. These tests were evaluated using the more conservative *p* < 0.001 uncorrected, given that the nominal FDR-adjusted *p* value threshold was estimated at *p* < 0.01.

The second research aim was addressed using zero-order Pearson correlations between ICC, TSA, and ICC-TSA conjunction indices, where significant group differences had been identified, and cognitive/emotional test scores (age- and education-adjusted z-scores for cognitive tests and raw scores of depression and anxiety scales). The statistical significance level was set to *p* < 0.001 uncorrected with nominal FDR-adjusted *p* < 0.003 (associations between TSA and test scores), *p* < 0.004 (associations between ICC and test scores), and *p* < 0.0008 (associations between coupling/uncoupling and test scores).

## Results

### Clinical, demographic, MRI, and neuropsychological characteristics of patients with chronic mTBI

Time post injury at the time of the MRI and neuropsychological evaluation averaged 18.4 months (*SD* = 11.7; see Table 1). The two groups (patients and HCs) were closely matched on age (mTBI mean = 41.73, *SD* = 17.1 years, HCs mean = 40.65, *SD* = 15.6 years), although the former group included a higher percentage of men (81.1% vs. 72%, *p* = 0.2), and had achieved lower formal education (mTBI mean = 11.72, *SD* = 3.8 years, HCs mean = 13.9, *SD* = 4.0 years, *p* = 0.01).

**Table 1:** Clinical, demographic, and neuropsychological information of patients with chronic mTBI

	*N* (%) / mean ± *SD*	Range
Age (years)	41.73 ± 17.1	19–65
Education (years)	11.72 ± 3.8	6 to 22
Gender: men (*n*/%)	30 (81.1%)	-
Trauma type:		
MVA (*n*/%)	20 (54.1%)	-
Fall (*n*/%)	17 (45.9%)	-
Months post injury	18.4 ± 11.7	5 to 60
GCS^1^	14.55 ± 0.9	13 to 15
CESD^1^	12.30 ± 8.8	0 to 35
CESD > 22 (n/%)	8 (21.6%)	-
STAI-B^1^	46.11 ± 10.5	32 to 75
STAI-B > 49 (n/%)	18 (48.6%)	-
Digits Forward^2^	− 0.67 ± 0.8†	− 2.6 to 1.2
Digits Reverse^2^	− 0.61 ± 0.7†	− 2.3 to 0.7
PM-Immediate^2^	− 1.10 ± 1.1†	− 3.7 to 1.0
PM-Delayed^2^	− 1.17 ± 1.0†	− 3.4 to 0.6
PM-Retention^2^	− 0.25 ± 1.1	− 2.5 to 2.1
PM-Recognition^2^	− 0.31 ± 2.0	− 2.4 to 1.2
TCF-Copy^2^	0.38 ± 0.8	− 1.9 tο 1.3
TCF-Memory^2^	− 0.12 ± 1.1	− 2.1 to 2.6
TMT-A^2^	0.60 ± 0.9	− 2.1 to 2.0
TMT-B^2^	0.38 ± 0.9	− 3.0 to 2.4
SVFT^2^	− 0.55 ± 0.9†	− 2.5 to 1.1
PhVFT^2^	− 1.14 ± 0.7†	− 3.0 to 0.4
WAIS-IV Matrices	− 1.01 ± 1.1†	− 2.8 to 2.0

Abbreviations;*GCS*, Glasgow Coma Scale; *CESD*, Center for Epidemiological Studies Depression scale; *STAI-B*, State-Trait Anxiety Inventory Form Y (Part B-Trait Anxiety); *MVA*, Motor Vehicle Accident; *TCF*, Taylor Complex Figure Test; *PM*, passage memory; *TMT*, Trail Making Test (part A and part B); *SVFT*, Semantic Verbal Fluency Test; *PhVFT*, Phonemic Verbal Fluency Test; *WAIS-IV*, Wechsler Adult Intelligence Scales. †Significant deviation from age- and education-adjusted population mean (p =<.001); ^1^Raw scores; ^2^Age- and education-adjusted z scores

Clinically significant depression or anxiety symptoms (according to the corresponding clinically validated cutoff scores on the CESD and STAI-B scales) were noted in 21.6% and 48.6% of the patients, respectively (see Table 1). Deficits in the domains of episodic memory or attention control and executive function (as indicated by performance > 1.5 *SD* below the national norms on at least two relevant cognitive tasks) were present in 24.3% and 32.4%, respectively (see Supplementary Table 1) of the patients. On individual tests, patient average performance was below 1 *SD* from the population mean on immediate (*z* =  − 1.10, *SD* = 1.1, *p* < 0.001) and delayed passage recall (*z* =  − 1.17, *SD* = 1.0, *p* < 0.001), phonemic verbal fluency (*z* =  − 1.14, *SD* = 0.7, *p* < 0.001), and WAIS-IV matrices (*z* =  − 1.01, *SD* = 1.1, *p* < 0.001). However, the presence of any of the estimated neuropsychiatric manifestations did not correlate with evidence of contusion or DAI in either frontal or temporal lobes (all Spearman *ρ*’s < 0.2, *p* > 0.2).

### Brain hemodynamic and connectivity characteristics in patients with chronic mild TBI

As shown in Table 2, patients with chronic mTBI displayed significant hypoconnectivity in medial temporal regions bilaterally (anterior hippocampus and limbic temporal pole). Conversely, patients with chronic mTBI showed hyperconnectivity in parietal regions, namely the left superior parietal lobule (SPL) and right angular gyrus. Results remained significant after controlling for age. Differences between mTBI and HC groups on TSA-derived indices did not reach significance.

**Table 2:** Regions displaying significantly higher or lower FC in patients with chronic mTBI as compared to HCs

ROI label (Schaefer atlas)	Anatomical region	MNI coordinates (x, y, z)	*p* value
*mTBI* > *Healthy Controls*			
LH Dorsal Attention Post-7	Superior Parietal Lobule	− 16, − 72, 54	0.001
RH parietal (DMN 1)	Angular gyrus	46, − 70, 28	0.0007
*mTBI* < *Healthy Controls*			
LH Limbic TP (1)	Parahippocampal gyrus	− 30, − 6, − 40	0.00003
LH Limbic TP (3)	Temporal pole	− 28, 10, − 34	0.00002
RH Limbic TP (3)	Parahippocampal gyrus	26, − 10, 32	0.00001
RH aHIP	Anterior hippocampus	30, − 12, 18	0.0002
LH aHIP	Anterior hippocampus	− 27, − 12, 19	0.001

*TP*, temporal pole; *DMN*, default mode network; *RH/LH*, right/left hemisphere; *aHIP*, anterior hippocampus. Reported results were significant at *p*<.001 uncorrected and exceeded the FDR-adjusted threshold of *p*<.01

### Association of brain hemodynamic characteristics with neuropsychiatric measures in patients with chronic mTBI

**Fig. 1:**
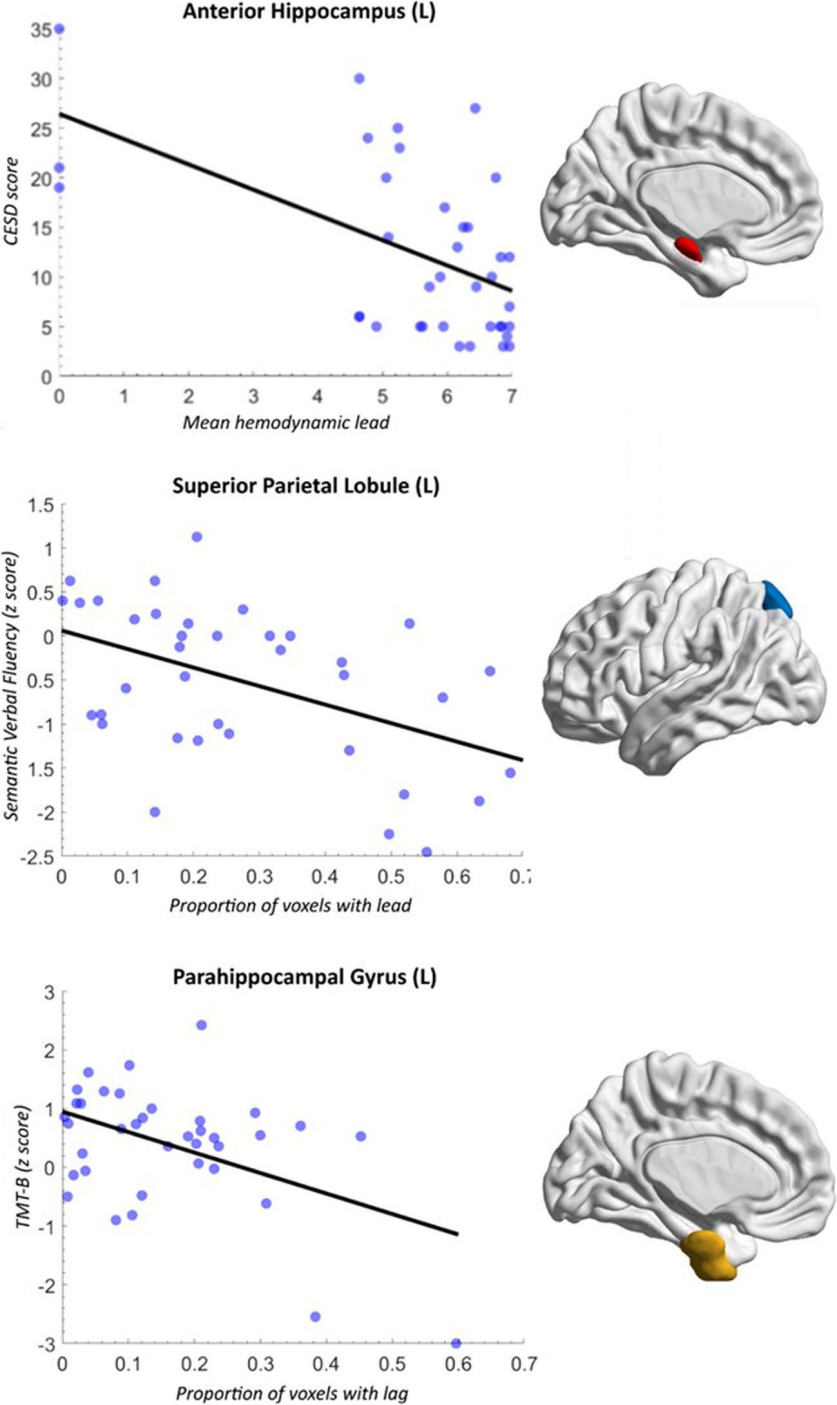
Scatterplots illustrating the association between hemodynamic indices and neuropsychiatric test scores (left-hand column) in the regions displayed on the right-hand side

As shown in Table 3 and Fig. 1, average hemodynamic lead in the left anterior hippocampus (which shows hypoconnectivity in mTBI) negatively correlated with depression (*r* =  − 0.530, *p* = 0.0006) and anxiety scores (*r* =  − 0.484, *p* = 0.002). The percentage of voxels displaying hemodynamic lag in the left anterior parahippocampal gyrus (which also showed hypoconnectivity in mTBI) negatively correlated with age- and education-adjusted scores on TMT-B (*r* =  − 0.461, *p* = 0.004). Moreover, the percentage of voxels displaying hemodynamic lead in the left SPL, which showed hyperconnectivity in mTBI, negatively correlated with semantic fluency (*r* =  − 0.474, *p* = 0.002). Results remained significant after controlling for age. Associations between ICC and emotional/cognitive test scores did not exceed *p* = 0.01 (uncorrected) or FDR-adjusted *p* < 0.02.

**Table 3:** Correlations between brain hemodynamics and neuropsychiatric measures in patients with chronic mild TBI

Region	Measure			Neuropsychological index	*r* (*p* value)
LH anterior hippocampus*	Hemodynamic lead		CESD		− 0.530 (0.0006)
LH anterior hippocampus*	Hemodynamic lead		STAI-B		− 0.484 (0.002)
LH parahippocampal gyrus*	Hemodynamic lag		TMT-B		− 0.461 (0.004)
LH Superior Parietal Lobule**	Hemodynamic lead	Semantic Fluency			− 0.474 (0.002)

Regions that showed hypoconnectivity (*) or hyperconnectivity (**) in mTBI as compared to HC; *TMT-B*, Trail Making Test (part B); *CESD*, Center for Epidemiological Studies Depression scale; *STAI-B*, State-Trait Anxiety Inventory Form Y (part B-Trait Anxiety). Reported results were significant at the FDR-adjusted threshold of *p*<.003

### Association of brain hemodynamics-connectivity coupling or uncoupling with neuropsychological measures in patients with chronic mTBI

**Fig. 2:**
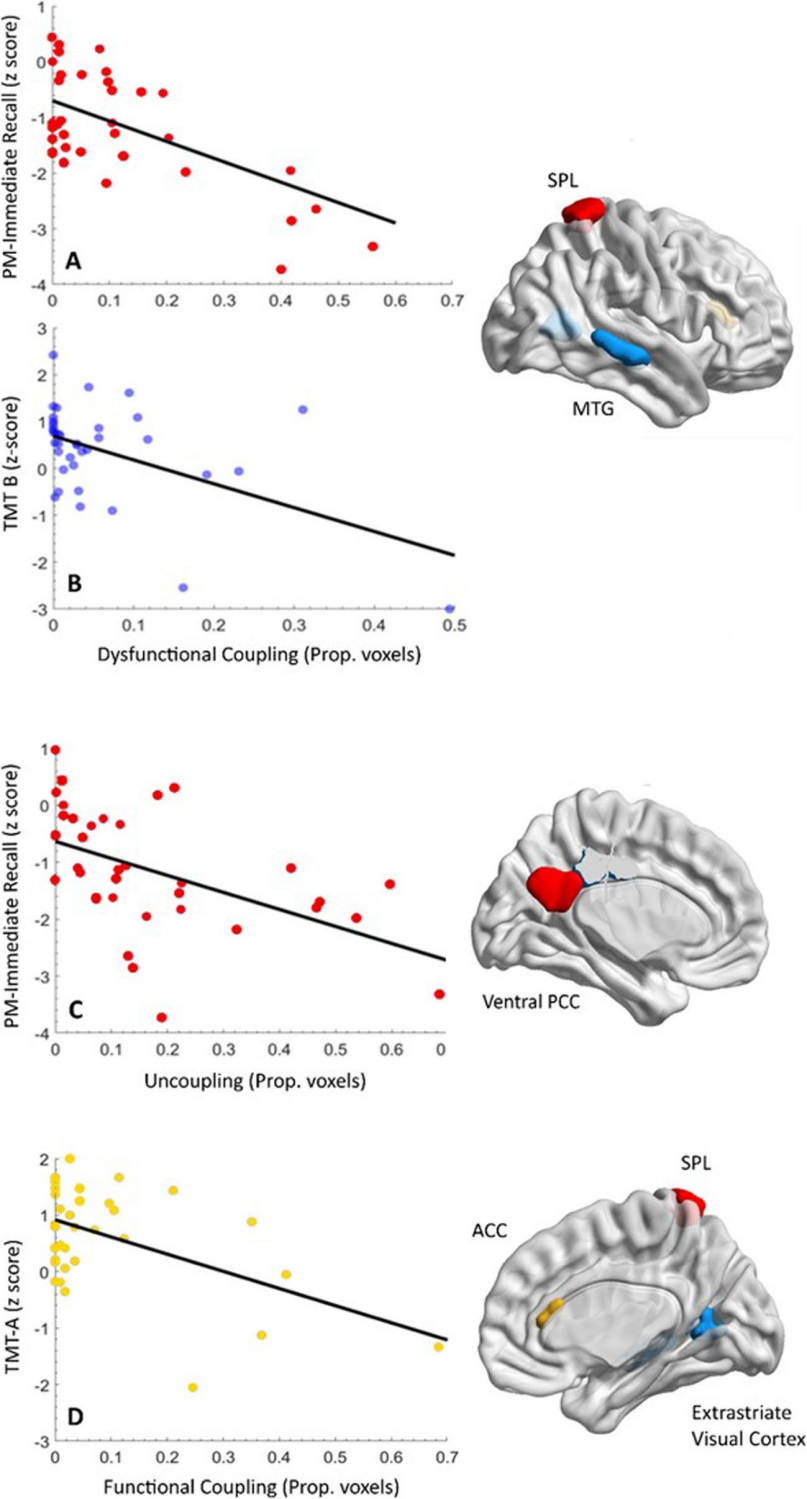
Scatterplots illustrating the association of indices of hemodynamics-FC coupling with neuropsychological test scores (left-hand column) in the regions displayed on the right-hand side

TSA-ICC conjunction indices can be categorized as “functional,” i.e., involving relatively higher regional FC and faster perfusion, or “dysfunctional,” involving relatively lower FC and slower perfusion. Correlations of TSA-ICC conjunction indices with emotional/cognitive status measures did not reach significance in any of the ROIs where significant group differences in regional FC were noted. In exploratory analyses, however (thresholded at *p* < 0.001, uncorrected), several interesting trends were noted (see Table 4 and Fig. 2).

**Table 4:** Associations of indices of regional hemodynamic-connectivity coupling or uncoupling with cognitive measures among patients with chronic mTBI

ROI label (Schaefer atlas)	Anatomical region	MNI coordinates (x, y, z)	Neuropsycho-logical index	*r* (*p* value)
*“Dysfunctional” coupling with low connectivity (hemodynamic lag* + *low ICC)*				
LH_Default_pCunPCC_2	Ventral PCC	− 6, − 54, 28	PM-Immediate	− 0.52 (0.001)
RH_DorsAttn_Post_10	SPL	22, − 48, 70	PM-Immediate	− 0.53 (0.0006)
*“Functional” coupling with high connectivity (hemodynamic lead* + *high ICC)*				
RH_Default_PFCdPFCm_3	ACC	6, 28, 16	TMT-A	− 0.50 (0.001)
*Uncoupling with high connectivity (Hemodynamic lag* + *High ICC)*				
RH_Vis_10	Extrastriate visual cortex	22, − 60, 8	TMT-B	− 0.50 (0.001)
RH_Default_Temp_4	MTG	62, − 26, − 6	TMT-B	− 0.50 (0.001)

*SPL*, superior parietal lobule; *PCC*, posterior cingulate cortex; *ACC*, anterior cingulate cortex; *MTG*, middle temporal gyrus; *PM*, passage memory; TMT, Trail Making Test (part A and part B). Results are thresholded at *p* < 0.001 uncorrected.

As shown in Table 4, *“dysfunctional coupling”* (hemodynamic lag + low ICC) of posterior DMN areas (left ventral posterior cingulate cortex (PCC)) and right SPL is negatively correlated with passage memory-immediate recall (*r* =  − 0.52, *p* = 0.001 and *r* = -0.53, *p* = 0.0006, respectively). *“Functional coupling”* (hemodynamic lead + high ICC) of right anterior DMN areas (right anterior cingulate cortex (ACC)) correlates negatively with visuomotor coordination, measured by TMT-A (*r* =  − 0.50, *p* = 0.001),

*Uncoupling* (hemodynamic lag + high ICC) in the medial extrastriate cortex and middle temporal gyrus (MTG) in the right hemisphere correlates negatively with cognitive flexibility, measured by TMT- B (*r* =  − 0.50, *p* = 0.001).

## Discussion

The main results of the current study can be summarised as follows:

- There is hypoconnectivity of bilateral hippocampi and parahippocampal gyri in patients with chronic mTBI as compared to the age- and gender-matched HCs group. The slower perfusion of the left anterior hippocampus was associated with relatively higher self-reported symptoms of depression and anxiety, while slower perfusion of the left parahippocampal gyrus was associated with relatively lower cognitive flexibility scores.
- “Functional” hemodynamic-connectivity coupling (high connectivity accompanied by hemodynamic lead) of the right ACC was significantly associated with lower performance on visuomotor coordination speed, while “dysfunctional” coupling (low connectivity and hemodynamic lag) of the left PCC and right SPL was associated with lower scores on immediate passage memory.
- Uncoupling (high connectivity and hemodynamic lag) of the right medial extrastriate cortex and middle temporal gyrus was negatively associated with cognitive flexibility.

### Relationship of hemodynamic and connectivity disturbances of limbic areas to anxiety, depression, and cognitive flexibility

In the current study, anterior limbic areas of patients with mild TBI, such as the hippocampus and parahippocampal gyrus, showed decreased FC compared to HCs, probably related to the increased vulnerability of these structures to head trauma [58]. Indeed, post traumatic lesions of the temporal lobes were found in many patients with chronic mild TBI in our study (32%) (see Supplementary Table 1). According to our results, slower perfusion in the hippocampus was associated with more severe anxiety and depression symptoms. This finding is potentially clinically significant in view of the high incidence of self-reported anxiety and depression symptoms in the current sample (48.6% and 21.6%, respectively; see Table 1), indicating impaired emotion regulation mechanisms. This is consistent with the view that the hippocampus plays a key role in downregulating the Hypothalamic–Pituitary–Adrenal (HPA) axis [59] and is in agreement with findings of reduced perfusion in bilateral parahippocampal cortex in patients with major depressive disorder (MDD) [60]. Only few studies have systematically examined hippocampal perfusion in mTBI with and without comorbid depressive symptomatology, and the results seem inconclusive. For instance, similar hippocampal perfusion was found among patients with MDD and patients with acute mTBI with or without depressive symptoms [61]. The present results highlight the role of hippocampal dysfunction in impaired emotion regulation, which may, in turn, trigger symptoms of anxiety and depression. It is possible that impaired hippocampal FC may, in some cases, be accompanied by reduced regional perfusion (leading to exacerbation of anxiety and depression symptoms) and in others by hyperperfusion as a compensatory mechanism to regulate heightened anxiety symptomatology elicited by mTBI. Future studies need to further elucidate the neurophysiological mechanisms involved in depression and anxiety regulation in these patients.

In the present study, the parahippocampal gyri of patients with chronic mTBI presented lower functional connectivity compared to HCs. Even more, slower perfusion of the left parahippocampal gyrus was associated with lower cognitive flexibility scores (as measured by the Trail Making Test-part B). This supports the notion that medial temporal lobe areas, including the hippocampus and parahippocampal cortex, are heavily involved in both cognitive and emotional symptomatology after mTBI. This finding might be explained by the increased vulnerability of the parahippocampal gyrus in brain trauma and the essential role of hippocampal neurogenesis in cognitive flexibility [58, 62]. In fact, studies report both beneficial and pathological effects of neurogenesis after mTBI, and various factors can account for this heterogeneity, such as the degree and region of injury, pathoanatomic functions, and diverse experimental procedures of TBI generation [63]. Moreover, newly formed neurons with aberrant anatomic characteristics in the dentate gyrus were found in mice after induced mTBI [64]. This could probably be related to poor network integration and impaired performance in pattern separation tasks, which is a core feature of cognitive flexibility [65]. Furthermore, pattern separation is a cognitive skill that may also be involved in anxiety regulation as it is essential for discrimination between safe and threatening contexts [66], while it is also a potential sign of impaired hippocampal adult neurogenesis in MDD [67]. Given the high comorbidity of mTBI with depressive and anxiety symptomatology and the role of pattern separation in the development of such symptoms, future studies should further examine its mechanisms and associations with mental health outcomes.

### Relationship of hemodynamic connectivity coupling/uncoupling of DMN areas with neurocognitive indices

We examined two types of hemodynamic connectivity coupling in this study. “Functional coupling” (hemodynamic lead and high ICC, interpreted as fast perfusion with hyperconnectivity) and “dysfunctional coupling” (hemodynamic lag and low ICC, interpreted as slow perfusion and hypoconnectivity). Notably, associations between each type of coupling with neurocognitive measures were mainly found in regions of the DMN. It should be noted that these should be treated as tentative given the exploratory nature of the analyses performed on the entire set of ROIs, types of coupling index, and cognitive/emotional scores and, as a consequence, medium-size correlations significant at *p* = 0.001 uncorrected, failed to survive FDR correction.

“Functional” hemodynamic-connectivity coupling in the right ACC was significantly associated with lower attention and visuomotor coordination performance. This could be explained by the involvement of ACC in attention regulation processes. Alterations of connectivity in the attention network, e.g., alterations in connectivity between ACC and posterior portions of the DMN, such as the precuneus, have been implicated in symptoms of impaired attention/concentration in persons with attention deficit hyperactivity disorder (ADHD) [68]. Even more, a significant relationship between ADHD symptomatology and mTBI has been reported [69, 70]. In addition, increased connectivity of bilateral ACC with the precuneus, occipital, and somatosensory regions was demonstrated in veterans with mTBI [71], suggesting a compensatory mechanism of the brain necessary to support cognitive functioning. Increased ACC coupling in our study may not be sufficient to support performance on TMT-A, and thus, there may be a disproportionate increase in functional coupling of ACC with insufficient improvement of TMT-A performance. The fact that there is no significant difference in TMT-A scores between patients and HCs may indicate the involvement of other regions to support performance on this task.

Dysfunctional coupling in the left ventral PCC and SPL (both components of the posterior DMN) was associated with lower scores on immediate passage memory. PCC has been assumed to play a vital role in memory processes due to its strong connections with the entorhinal cortex and parahippocampal gyrus [72]. Dysfunctional PCC perfusion and functional connectivity have long been observed in diseases with predominant memory system dysregulation, such as Alzheimer’s disease [73]. The correlation of dysfunctional coupling of PCC with memory scores among patients with chronic mTBI in our study is probably related to increased vulnerability of that region after mild injury [74], leading to decreased perfusion and abnormal functional connectivity, as has also been shown in previous studies [75, 76]. This is the first study that demonstrates a relationship between functional coupling in ACC and dysfunctional coupling in PCC with lower cognitive performance in chronic mTBI patients. These coupling differences may indicate a compensatory role of ACC in the face of PCC vulnerability and dysfunction after brain trauma, corroborated by other fMRI studies [58, 72].

Functional and hemodynamic connectivity coupling in chronic mTBI patients of our study could be explained by the molecular mechanisms related to induced neurogenesis and angiogenesis after brain injury [77, 78]. Even more, a potential role of the vascular system in the augmentation of neurogenesis after TBI has also been suggested, demonstrating a close interaction of the two systems necessary for coupling [79]. Additionally, newborn neuronal cells may present aberrant characteristics after mTBI [63, 64], potentially leading to dysfunctional coupling.

Finally, uncoupling (hemodynamic lag and high ICC, interpreted as slow perfusion and hyperconnectivity) of the right medial extrastriate cortex and posterior MTG in the right hemisphere was negatively correlated with cognitive flexibility. While the former region is part of the visual association cortex, the latter is part of the tertiary, polymodal cortex, with limited data in humans on the role of these areas in the right hemisphere [80]. The present preliminary data suggest that impaired neurovascular processes in these areas may represent a specific vulnerability in the chronic phase of mTBI, which is reflected primarily in complex tasks involving visual scanning and eye-hand coordination under increased cognitive demands (i.e., when applying a dual strategy requiring constant switching between numbers and letters).

Although uncoupling between heightened neuronal metabolic demands and simultaneous decreased blood flow has been noted in acute rodent models of ΤΒΙ [81], the investigation of neurovascular uncoupling in the chronic phase of TBI and its association with neurocognitive indices has been limited. There are various possible explanations for neurovascular uncoupling in TBI, such as the faulty regulation mechanisms of CBF leading to an imbalance between impaired blood flow and increased metabolic demands [82]. Indeed, the upregulation of endothelin, a strong brain vasoconstrictor, has been widely noted after TBI and has been linked to worse neurocognitive outcomes [83]. Significantly reduced global or local hypoperfusion was detected in patients with chronic mTBI [11–14] and contributed to cognitive decline and poor psychoemotional outcomes [14]. Evidence of neurovascular uncoupling, contributing to cognitive impairment, has also been described in Alzheimer’s disease, in addition to global hypoperfusion that is thought to precede significant neurodegeneration [84].

### Limitations

There are several limitations in the current cross-sectional study, including the small sample size and gender imbalance. In addition, cognitive performance could be affected by measurement errors in cognitive test scores and also by emotional disturbances, particularly depression and anxiety. Moreover, the present results represent predominantly males who have suffered TBI and may not be representative of women and other types of traumatic injury. This bias follows the significantly higher incidence of mild/moderate TMI due to MVA or falls among working-age men resulting from engagement in high-risk occupations and activities/behaviors [85]

Furthermore, while some previous studies have discovered hemodynamic differences between patients with chronic mTBI and HCs, TSA analysis failed to reveal such differences. These discrepant findings may reflect the different methods of extracting hemodynamic data (DSC-MRI or ASL perfusion MRI) as well as heterogeneity in injury severity and time from injury among studies.

Finally, although ICC is a promising technique for uncovering functional connectivity without the definition of a priori ROIs, it does not pinpoint the specific connectivity alterations between regions. Subsequent studies should utilize ICC to detect regions exhibiting altered connectivity and jointly employ ROI-based methods to further delineate their connectivity patterns [86].

## Conclusions

To our knowledge, this is the first study that examined the association of both hemodynamic and connectivity disturbances and their coupling/uncoupling changes with neurocognitive and mental health indices in the chronic phase of mTBI by analyzing rs-fMRI data. The findings demonstrate a role of perfusion of the hippocampus in the modulation of mental health indices after mTBI, coupling changes in anterior and posterior DMN indicating potential compensatory mechanisms, and uncoupling in the right medial extrastriate cortex and posterior MTG, which might be explained by complex interactions of the neurovascular components. Future studies will need to further validate our results by adopting a longitudinal approach, examining coupling differences over time, and investigating possible genetic predisposition, molecular mechanisms, and microstructural changes underlying these outcomes.

These findings pave the way for future investigations, and they are clinically relevant in various domains. Firstly, the unraveling of neuroimaging biomarkers and their association with the observed behavioral and neurocognitive deficits may lead to a better understanding of the pathophysiological mechanisms underlying mTBI. Secondly, current treatment choices focus on addressing clinical symptoms instead of underlying pathophysiology. A better understanding of neurovascular processes in mTBI can lead to optimized and personalized treatment decisions and efficacy, given that different patterns of neurovascular dysfunction may be associated with specific observed impairments. The incorporation of neurovascular indices in routine clinical practice of patients with complicated mTBI may also contribute to intervention studies as potential predictors of response to treatment.

### Supplementary Information

Below is the link to the electronic supplementary material.

Supplementary file1 (DOCX 200 KB)

## Data Availability

The computed metrics derived from resting-state fMRI recordings will be available upon request**.**

## Declarations

### Relation of this study to previous publications

The group of chronic mTBI patients in this study was similar, but not identical, to that of the reference 28, while the battery of neuropsychological tests and the parameters of the brain MRI sequences (including rs-fMRI), as well as the initial fMRI data preparation steps are the same with those of the reference 28.
