# Supplementary material for: Functional connectivity-hemodynamic (un)coupling changes in chronic mild brain injury are associated with mental health and neurocognitive indices: a resting state fMRI study
Source: Neuroradiology. 2024 Apr 12;66(6):985–98. doi: 10.1007/s00234-024-03352-9 (PMC11133187; doi:10.1007/s00234-024-03352-9)
Supplement: Supplementary file 1 — Supplementary file1 (DOCX 200 KB) [file 234_2024_3352_MOESM1_ESM.docx]

**Functional connectivity/ hemodynamic (un)coupling changes in chronic mild brain injury are associated with mental health and neurocognitive indices: A resting state fMRI study.**

**SUPPLEMENTARY MATERIAL**

1. **MATERIALS AND METHODS**
   1. ***MRI data preprocessing and denoising***

Initial data preparation steps are in line with previous work of our team on data from the same MRI system [1-5]. Each BOLD time series consisted of 150 dynamic volumes (the first five were ignored in all subsequent analyses). Preprocessing steps included slice-time correction, realignment, segmentation of structural data, normalization into standard stereotactic Montreal Neurological Institute (MNI) space and spatial smoothing using a Gaussian kernel of 8 mm full-width at half-maximum using SPM8. As functional connectivity and temporal changes measured by the BOLD signal are affected by head motion in the scanner, we accounted for motion artifact detection and rejection using the artifact detection tool (ART; http://www.nitrc.org/projects/ artifact detect). The first five principal components of CSF regions were regressed out of the signal as well as their first order derivatives. These steps were completed using CompCor implemented within the CONN preprocessing module [6] and executed in MATLAB. The fMRI time series were detrended and bandpass filtered in the 0.008-0.09 Hz range, to eliminate low frequency drift and high frequency noise [1-5]. Supplementary figure 1 depicts the anatomy of the venous mask used for extraction of the reference signal in TSA analyses. This is shown in free topological fashion as well as overlaid on standard T1 image.


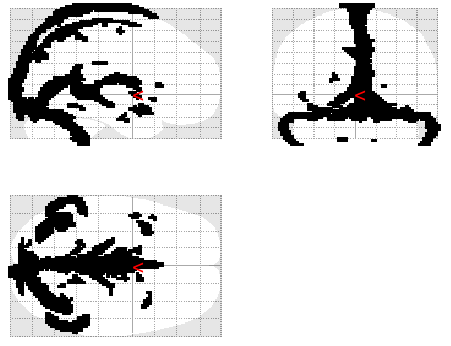


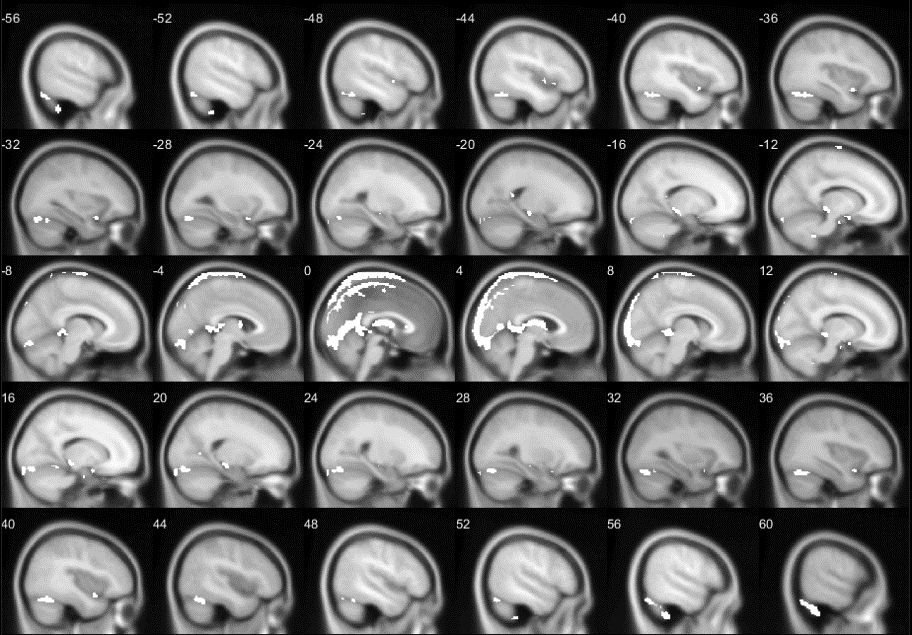


- 1. ***Voxel-wise functional connectivity***

Voxel-wise global connectivity was assessed through the Intrinsic Connectivity Contrast (ICC) an estimate of the degree of association between the time-series of a given voxel with all the remaining voxels in the brain (in the present study voxels included in all 232 regions of the Schaefer atlas) [7]. ICC is based on the graph-theoretical measure of degree. Degree signifies the number of nodes connected to each node, while the calculation of ICC, on a weighted graph, takes into account the connectivity strengths of all connections present for each node. Specifically, a voxel’s ICC value is computed as the mean of that voxel’s time series correlation values with all other voxels’ time series, squared. The explicit calculation of full voxel-to-voxel functional connectivity matrices in fMRI datasets such as the present is computationally prohibitive mostly due to software RAM usage limitations. MATLAB code for the calculation of ICC maps using Singular Value Decomposition, similar to the implementation found in CONN [6] and is freely available online [8; <https://www>.mathworks.com/matlabcentral/fileexchange/68248-intrinsic_connectivity_contrast, MATLAB Central File Exchange].

Two FC indices were computed per ROI and participant: (i) percentage of voxels with ICC values greater than the 75^th^ percentile of all cortical voxels of a given participant and (ii) percentage of voxels with ICC values smaller than the 25^th^ percentile of all cortical voxels of a given participant. Additionally, two sets of binary ICC masks were computed per ROI and participant: in one all voxels with ICC values exceeding the above-mentioned 75^th^ percentile were labeled with 1 (and the remainder were assigned the value of 0); in the second all voxels with ICC values lower than the corresponding 25^th^ percentile value of the global subject-specific ICC map were labeled with 1 (and the remainder were assigned the value of 0).

1. **RESULTS**

***Clinical, demographic, MRI and neuropsychological characteristics of mTBI patients***

As shown in Table 1, MRI scans containing small posttraumatic gliotic areas due to contusions (≤ 3 cm in extent) were found in 40.5% of the patients (in 7/37 patients in the left temporal lobe, in 7/37 patients in the right temporal lobe, in 7/37 patients in the right frontal lobe, and 8/37 patients in the left frontal lobe). Evidence of ≤ 3 chronic hemorrhagic Diffuse Axonal Injuries (DAIs) was found in 27% of the patients (in 4/37, 1/37, 6/37, and 9/37 patients in the right temporal, left temporal, right frontal, or left frontal lobes, respectively).

**Table 1** Individual demographic and clinical data, conventional MRI and neuropsychiatric manifestations in chronic mTBI patients.

| **ID** | **Age (years)** | **Edu** | **GCS** | **TPI** | **Lesion** | **Depression symptoms** | **Anxiety symptoms** | **Episodic Memory deficits** | **Executive/ Attention deficits** |
| --- | --- | --- | --- | --- | --- | --- | --- | --- | --- |
| 1 | 47 | 12 | 13 | 18 | C (L/RT, L/RF), DAI (RF) | -- | -- | -- | -- |
| 2 | 43 | 6 | 15 | 6 | -- | -- | -- | -- | -- |
| 3 | 45 | 6 | 13 | 5 | C (L/RT, LF) | yes | -- | -- | yes |
| 4 | 19 | 12 | 15 | 36 | -- | -- | -- | -- | yes |
| 5 | 50 | 12 | 15 | 11 | C (L/RT, L/RF) | -- | yes | -- | -- |
| 6 | 64 | 6 | 14 | 15 | C (RT, L/RF) | -- | -- | yes | yes |
| 7 | 63 | 6 | 15 | 30 | DAI (L/RF) | -- | -- | -- | -- |
| 8 | 48 | 7 | 14 | 19 | -- | -- | yes | -- | -- |
| 9 | 64 | 17 | 15 | 12 | -- | -- | yes | yes | -- |
| 10 | 39 | 12 | 14 | 10 | C (RF), DAI (RF) | -- | -- | -- | -- |
| 11 | 50 | 13 | 15 | 20 | C (RF) | -- | -- | yes | yes |
| 12 | 41 | 6 | 15 | 31 | -- | -- | -- | -- | -- |
| 13 | 24 | 12 | 14 | 29 | DAI (L/RF) | -- | -- | -- | -- |
| 14 | 21 | 15 | 14 | 14 | DAI (RF) | -- | yes | -- | yes |
| 15 | 59 | 12 | 15 | 36 | -- | -- | yes | -- | -- |
| 16 | 47 | 8 | 15 | 24 | -- | -- | -- | yes | -- |
| 17 | 65 | 16 | 15 | 15 | -- | yes | yes | -- | -- |
| 18 | 46 | 22 | 15 | 16 | -- | -- | yes | -- | yes |
| 19 | 65 | 6 | 15 | 15 | DAI (L/RF) | yes | yes | -- | -- |
| 20 | 19 | 12 | 15 | 14 | -- | -- | yes | -- | -- |
| 21 | 22 | 12 | 15 | 12 | -- | -- | -- | -- | yes |
| 22 | 30 | 14 | 14 | 60 | C (LT, LF) | -- | -- | -- | yes |
| 23 | 22 | 16 | 15 | 12 | C (LT) | -- | -- | -- | -- |
| 24 | 18 | 12 | 15 | 11 | C (L/RF) | -- | yes | -- | -- |
| 25 | 64 | 12 | 15 | 36 | -- | -- | yes | -- | -- |
| 26 | 36 | 6 | 15 | 16 | C (RF) | yes | yes | -- | -- |
| 27 | 64 | 12 | 15 | 16 | C (RT) | yes | yes | yes | yes |
| 28 | 21 | 15 | 14 | 13 | C (LT), DAI (LT, L/RF) | -- | -- | -- | yes |
| 29 | 19 | 12 | 15 | 36 | -- | -- | -- | yes | yes |
| 30 | 56 | 14 | 15 | 12 | -- | -- | yes | -- | -- |
| 31 | 65 | 17 | 15 | 29 | -- | yes | yes | -- | -- |
| 32 | 18 | 12 | 14 | 6 | C (RT, L/RF), DAI (LT, L/RF) | -- | -- | yes | -- |
| 33 | 45 | 11 | 14 | 6 | C (L/RT) | yes | yes | -- | -- |
| 34 | 28 | 15 | 15 | 11 | DAI (LT, L/RF) | -- | -- | yes | -- |
| 35 | 47 | 12 | 15 | 8 | -- | -- | yes | -- | -- |
| 36 | 52 | 12 | 15 | 14 | -- | yes | yes | -- | -- |
| 37 | 18 | 12 | 13 | 7 | DAI (L/RT) | -- | -- | yes | yes |

Abbreviations; L: Left and R: Right hemisphere, T: Temporal, F: Frontal, GCS: Glasgow Coma Scale, DAI: 1-3 chronic hemorrhagic foci resulting from diffuse axonal injuries, C: gliotic areas due to contusions<=3cm, Edu: Education in years, TPI: Time post injury in months.

**REFERENCES**

1. Antypa D, Simos NJ, Kavroulakis E, Bertsias G, Fanouriakis A, Sidiropoulos P, Boumpas D, Papadaki E. Anxiety, and depression severity in neuropsychiatric SLE are associated with perfusion and functional connectivity changes of the frontolimbic neural circuit: a resting-state f(unctional) MRI study. Lupus Sci Med. 2021 Apr;8(1): e000473. doi: 10.1136/lupus-2020-000473.
2. Antypa D, Simos NJ, Panou T, Spyridaki E, Kagialis A, Kosteletou E, Kavroulakis E, Mastorodemos V, Papadaki E. Distinct hemodynamic and functional connectivity features of fatigue in clinically isolated syndrome and multiple sclerosis: accounting for the confounding effect of concurrent depression symptoms. Neuroradiology. 2023 Jun 10. doi: 10.1007/s00234-023-03174-1.
3. Kavroulakis E, Simos NJ, Maris TG, Zaganas I, Panagiotakis S, Papadaki E. Evidence of Age-Related Hemodynamic and Functional Connectivity Impairment: A Resting State fMRI Study. Front Neurol. 2021 Mar 23; 12:633500. doi: 10.3389/fneur.2021.633500.
4. Simos NJ, Manikis GC, Papadaki E, Kavroulakis E, Bertsias G, K. Marias K. Machine Learning Classification of Neuropsychiatric Systemic Lupus Erythematosus Patients Using Resting-State fMRI Functional Connectivity 2019 IEEE International Conference on Imaging Systems and Techniques (IST), Abu Dhabi, United Arab Emirates, 2019, pp. 1-6, doi: 10.1109/IST48021.2019.9010078.
5. Simos NJ, Dimitriadis SI, Kavroulakis E, Manikis GC, Bertsias G, Simos P, Maris TG, Papadaki E. Quantitative Identification of Functional Connectivity Disturbances in Neuropsychiatric Lupus Based on Resting-State fMRI: A Robust Machine Learning Approach. Brain Sci. 2020 Oct 25;10(11):777. doi: 10.3390/brainsci10110777.
6. Whitfield-Gabrieli S, Nieto-Castanon A. Conn: a functional connectivity toolbox for correlated and anticorrelated brain networks. Brain Connect. 2012;2(3):125-41. doi: 10.1089/brain.2012.0073.
7. Schaefer A, Kong R, Gordon EM, Laumann TO, Zuo XN, Holmes AJ, Eickhoff SB, Yeo BTT. Local-Global Parcellation of the Human Cerebral Cortex from Intrinsic Functional Connectivity MRI. Cereb Cortex. 2018 Sep 1;28(9):3095-3114. doi: 10.1093/cercor/bhx179.
8. Layden E. Intrinsic_connectivity_contrast (Internet). MATLABCentral File Exchange. 2020 (cited 2020 Apr 2). Available from: https://www.mathworks.com/matlabcentral/fileexchange/68248-intrinsic_connectivity_contrast
